# What Is Life—and How Do We Search for It in Other Worlds?

**DOI:** 10.1371/journal.pbio.0020302

**Published:** 2004-09-14

**Authors:** Chris P McKay

## Abstract

If there is life on distant worlds, how would we go about finding it?

I need a “tricorder”—the convenient, hand-held device featured on *Star Trek* that can detect life forms even from orbit. Unfortunately, we don't have a clue how a tricorder might work, since life forms don't seem to have any observable property that distinguishes them from inanimate matter. Furthermore, we lack a definition of life that can guide a search for life outside Earth. How can we find what we can't define? An answer may lie in the observation that life uses a small, discrete set of organic molecules as basic building blocks. On the surface of Europa and in the subsurface of Mars, we can search for alien but analogous patterns in the organics.

## Life As We Know It

The obvious diversity of life on Earth overlies a fundamental biochemical and genetic similarity. The three main polymers of biology—the nucleic acids, the proteins, and the polysaccarides—are built from 20 amino acids, five nucleotide bases, and a few sugars, respectively. Together with lipids and fatty acids, these are the main constituents of biomass: the hardware of life (Lehninger 1975, p 21). The DNA and RNA software of life is also common, indicating shared descent (Woese 1987). But with only one example of life—life on Earth—it is not all that surprising that we do not have a fundamental understanding of what life is. We don't know which features of Earth life are essential and which are just accidents of history.

Our lack of data is reflected in our attempts to define life. Koshland (2002) lists seven features of life: (1) program (DNA), (2) improvisation (response to environment), (3) compartmentalization, (4) energy, (5) regeneration, (6) adaptability, and (7) seclusion (chemical control and selectivity). A simpler definition is that life is a material system that undergoes reproduction, mutation, and natural selection (McKay 1991). Cleland and Chyba (2002) have suggested that life might be like water, hard to define phenomenologically, but easy to define at the fundamental level. But life is like fire, not water—it is a process, not a pure substance. Such definitions are grist for philosophical discussion, but they neither inform biological research nor provide a basis for the search for life on other worlds.

The simplest, but not the only, proof of life is to find something that is alive. There are only two properties that can determine if an object is alive: metabolism and motion. (Metabolism is used here to include an organism's life functions, biomass increase, and reproduction.) All living things require some level of metabolism to remain viable against entropy. Movement (either microscopic or macroscopic) in response to stimuli or in the presence of food can be a convincing indicator of a living thing. But both metabolism (fire) and motion (wind) occur in nature in the absence of biology.

The practical approach to the search for life is to determine what life needs. The simplest list is probably: energy, carbon, liquid water, and a few other elements such as nitrogen, sulfur, and phosphorus (McKay 1991). Life requires energy to maintain itself against entropy, as does any self-organizing open system. In the memorable words of Erwin Schrödinger (1945), “It feeds on negative entropy.” On Earth, the vast majority of life forms ultimately derive their energy from sunlight. The only other source of primary productivity known is chemical energy, and there are only two ecosystems known, both methanogen-based (Stevens and McKinley 1995; Chapelle et al. 2002), that rely exclusively on chemical energy (that is, they do not use sunlight or its product, oxygen). Photosynthetic organisms can use sunlight at levels below the level of sunlight at the orbit of Pluto (Ravens et al. 2000); therefore, energy is not the limitation for life. Carbon, nitrogen, sulfur, and phosphorus are the elements of life, and they are abundant in the Solar System. Indeed, the Sun and the outer Solar System have more than 10,000 times the carbon content of the bulk of Earth (McKay 1991). When we scan the other worlds of our Solar System, the missing ecological ingredient for life is liquid water. It makes sense, then, that the search for liquid water is currently the first step in the search for life on other worlds. The presence of liquid water is a powerful indication that the ecological prerequisites for life are satisfied.

**Figure 1:**
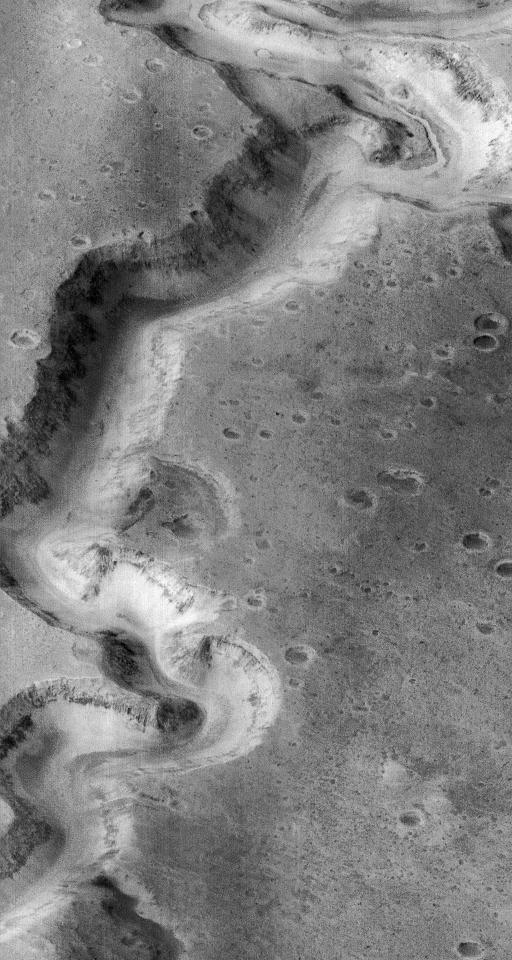
Water on Another World A Mars Global Surveyor image showing Nanedi Vallis in the Xanthe Terra region of Mars. The image covers an area 9.8 km ×18.5 km; the canyon is about 2.5 km wide. This image is the best evidence we have of liquid water anywhere outside the Earth. Photo credit: NASA/Malin Space Sciences.

Orbital images, such as the canyon in Figure 1, show clear evidence of the stable and repeated, if not persistent, flow of a low-viscosity fluid on Mars at certain times in its past history. The fluid was probably water, but the images could also suggest wind, ice, lava, even carbon dioxide or sulfur dioxide. Recently, results from the Mars Exploration Rover missions have shown that this liquid carried salts and precipitated hematite in concretions. The case for water, we could say, is tight.

On Jupiter's moon Europa, the cracks and icebergs on the surface of the ice indicate water beneath the ice, but not necessarily at the present time. Present water on Europa is indicated by the magnetic disturbance Europa makes as it moves through Jupiter's magnetic field, not unlike the way coins in the pocket of a passenger will set off an airport metal detector. Europa has a large conductor, and this is most likely a global, salty layer of water.

## Viking on Mars: Been There, Tried That

The Viking missions to Mars in the late 1970s were the first (and as yet, the only) search for life outside Earth. Each Viking conducted three incubation experiments to detect the presence of metabolism in the Martian soil. Each lander also carried a sophisticated Gas Chromatograph Mass Spectrometer for characterizing organic molecules. The results were unexpected (Klein 1978, 1999). There was a detectable reaction in two of the incubation experiments. In the “Gas Exchange” experiment, a burst of oxygen was released when the soil was exposed to water. The “Labeled Release” experiment showed that organic material was consumed, and that carbon dioxide was released concomitantly. In the Labeled Release experiment, this reaction ceased if the soil was first heated to sterilizing temperatures, but the reaction of the Gas Exchange Experiment persisted.

If considered alone, the Labeled Release results would be a plausible indication for life on Mars. However, the Gas Chromatograph Mass Spectrometer did not detect the presence of any organic molecules in the soil at level of one part per billion (Biemann 1979). It is difficult to imagine life without associated organic material, and this is the main argument against a biological interpretation of the Viking results (Klein 1999; but cf. Levin and Straat 1981). It is also unlikely that the oxygen release in the Gas Exchange experiment had a biological explanation, because the reaction was so rapid and persisted after heating. It is generally thought that the reactivity observed by the Viking biology experiments was caused by one or more inorganic oxidants present in the soil, and was ultimately produced by ultraviolet light in the atmosphere. Consistent with the apparently negative results of the Viking biology experiments, the surface of Mars also appears to be too dry for life. Indeed, conditions are such that liquid water is rare and transient, if it occurs at all (e.g., Hecht 2002).

## It's Life, Jim, but Not As We Know It

Table 1 shows a categorization of life as we have observed it. Using this diagram, we can speculate about how life might be different on Mars or Europa. At the bottom of the table, life is composed of matter—a reasonable assumption for now. Carbon and liquid water are the next level; this makes Mars and Europa likely candidates, because they have carbon and have, or have had, liquid water. Other worlds may have a different chemical baseline for life. The usual speculation in this area is that the presence of ammonia and silicon, rather than water and carbon, might be preconditions for life on other planets. Such speculation has yet to lead to any specific suggestions for experiments, or to new ways to search for such life, but this may just reflect a failure of human imagination rather than a fundamental limitation on the nature of life.

**Table 1:**
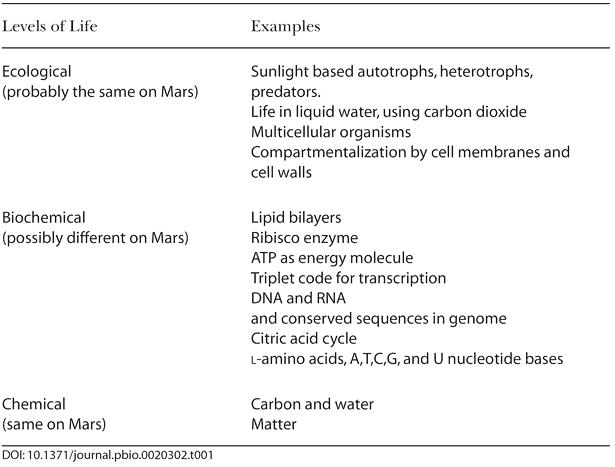
A Categorization of Structures That Comprise Terrestrial Life

Life on Mars is also likely to be the same at the top of the table: at the ecological level. Primary production in a Martian ecosystem is likely to be phototrophic, using carbon dioxide and water. Heterotrophs are likely to be present to consume the phototrophs and in turn to be consumed by predators. Darwinian evolution would result in many of the same patterns we see in ecosystems on Earth. While it may be similar at the top (ecological) and bottom (chemical) levels, life on Mars could be quite alien in the middle, in the realm of biochemistry. Pace (2001) has argued that alien biochemistry will turn out to be the same as biochemistry on Earth, because there is one best way to do things and that natural selection will ensure that life everywhere discovers that way. Only observation will tell if there is one possible biochemistry, or many.

Future missions to Mars might find microfossils in sedimentary rocks such as those at Meridiani Planum. Microbes don't readily form convincing fossils; the one exception may be the strings of magnetite formed by magnetotactic bacteria (Friedmann et al. 2001). As interesting as fossils might be, we could not be sure that a fossil found on Mars was not merely another example of Earth life. We know that rocks have come to Earth from Mars, and it is possible that such rocks could have carried life between the planets (Mileikowsky et al. 2000; Weiss et al. 2000). Finding fossil evidence for life on Mars does not demonstrate a second genesis in our Solar System.

## Finding a Way to Search for Alien Life

If we were to find organic material in the subsurface of Mars or on the ice of Europa, how could we determine whether it was the product of a system of biology or merely abiotic, organic material from meteorites or photochemistry? If this life were in fact related to Earth life, this should be easy to determine. We now have very sensitive methods, such as PCR and fluorescent antibody markers, for detecting life like us. This case would be the simplest to determine, but it would also be the least interesting. If the life turned out to be truly alien, then the probes specific to our biology would be unlikely to work. What, then, could we do to determine a biological origin?

The question is open and possibly urgent. On space missions already being planned, we may have the opportunity to analyze the remains of alien organics on the surface of Europa or frozen below ground on Mars. The instruments that will make this analysis must be designed in the next couple of years.

**Figure 2:**
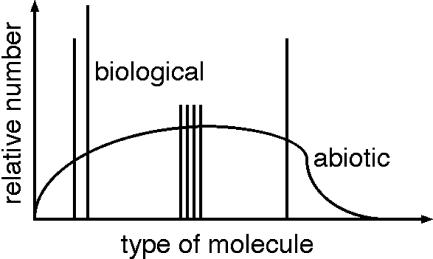
Comparison of Biogenic with Nonbiogenic Distributions of Organic Material Nonbiological processes produce smooth distributions of organic material, illustrated here by the curve. Biology, in contrast selects and uses only a few distinct molecules, shown here as spikes (e.g., the 20 l-amino acids on Earth). Analysis of a sample of organic material from Mars or Europa may indicate a biological origin if it shows such selectivity.

One approach appears promising. I call it the “Lego Principle.” It is based on the patterns of the molecules of life. Biological processes, in contrast to abiotic mechanisms, do not make use of the range of possible organic molecules. Instead, biology is built from a selected set. Thus, organic molecules that are chemically very similar to each other may have widely different concentrations in a sample of biological organics. An example of this on Earth is the 20 amino acids used in proteins and the use of the left-handed version of these amino acids. The selectivity of biological processes is shown schematically in Figure 2 by the distribution of spikes in contrast to a smooth, nonbiological distribution. General arguments of thermodynamic efficiency and specificity of enzymatic reactions suggest that this selectivity is required for biological function and is a general result of natural selection. Different life forms are likely to have different patterns, and at the very least we might find the mirror symmetry of life on Earth, with d- instead of l-amino acids.

This approach has immediate practical benefit in the search for biochemistry in the Solar System. Samples of organic material collected from Mars and Europa can be easily tested for the prevalence of one chirality of amino acid over the other. More generally, a complete analysis of the relative concentration of different types of organic molecules might reveal a pattern that is biological even if that pattern does not involve any of the familiar biomolecules. Interestingly, if a sample of organics from Mars or Europa shows a preponderance of D-amino acids, this would be evidence of life, and at the same time would show that this life was distinct from Earth life. This same conclusion would apply to any clearly biological pattern that was distinct from that of Earth life.

Organic material of biological origin will eventually lose its distinctive pattern when exposed to heat and other types of radiation, (examples of this include the thermal racemization of amino acids), but at the low temperatures in the Martian permafrost, calculations suggest that there has been no thermal alteration (Kanavarioti and Mancinelli 1990). An interesting question, as yet unanswered, is how long organic material frozen into the surface ice of Europa would retain a biological signature in the strong radiation environment.

On Europa, the organic material for our tests might be collected right from the dark regions on the surface. On Mars, there is ice-rich ground in the cratered southern polar regions (Feldmann et al. 2002), which presumably overlies deeper, older ice. The surprise discovery of strong magnetic fields in the southern hemisphere of Mars (Acuña et al. 1999; Connerney et al. 1999) indicates that the area may be the oldest undisturbed permafrost on that planet. Like the mammoths extracted from the ice in Siberia, any Martian microbes found in this ice would be dead, but their biochemistry would be preserved. From these biological remains, it would then be possible to determine the biochemical composition of, and the phylogenetic relationship between, Earth life and Martian life. We may then have, for the first time, a second example of life.

## References

[pbio-0020302-Acuna1] Acuña MH, Connerney JEP, Ness NF, Lin RP, Mitchell D (1999). Global distribution of crustal magnetism discovered by the Mars Global Surveyor MAG/ER experiment. Science.

[pbio-0020302-Biemann1] Biemann K (1979). The implications and limitations of the findings of the Viking Organic Analysis Experiment. J Mol Evol.

[pbio-0020302-Chapelle1] Chapelle FH, O'Neil K, Bradley PM, Methé BA, Ciufo SA (2002). A hydrogen-based subsurface microbial community dominated by methanogens. Nature.

[pbio-0020302-Cleland1] Cleland CE, Chyba CF (2002). Defining life. Orig Life Evol Biosph.

[pbio-0020302-Connerney1] Connerney JEP, Acuna MH, Wasilewski P, Ness NF, Reme H (1999). Magnetic lineations in the ancient crust of Mars. Science.

[pbio-0020302-Feldman1] Feldman WC, Boynton WV, Tokar RL, Prettyman TH, Gasnault O (2002). Global distribution of neutrons from Mars: Results from Mars Odyssey. Science.

[pbio-0020302-Friedmann1] Friedmann EI, Wierzchos J, Ascaso C, Winklhofer M (2001). Chains of magnetite crystals in the meteorite ALH84001: Evidence of biological origin. Proc Natl Acad Sci U S A.

[pbio-0020302-Hecht1] Hecht MH (2002). Metastability of liquid water on Mars. Icarus.

[pbio-0020302-Kanavarioti1] Kanavarioti A, Mancinelli RL (1990). Could organic matter have been preserved on Mars for 3.5 billion years?. Icarus.

[pbio-0020302-Klein1] Klein HP (1978). The Viking biological experiments on Mars. Icarus.

[pbio-0020302-Klein2] Klein HP (1999). Did Viking discover life on Mars?. Orig Life Evol Biosph.

[pbio-0020302-Koshland1] Koshland DE (2002). The seven pillars of life. Science.

[pbio-0020302-Lehninger1] Lehninger AL (1975). Biochemistry.

[pbio-0020302-Levin1] Levin GV, Straat PA (1981). A search for nonbiological explanation of the Viking labeled release life detection experiment. Icarus.

[pbio-0020302-McKay1] McKay CP (1991). Urey Prize lecture: Planetary evolution and the origin of life. Icarus.

[pbio-0020302-McKay2] McKay CP (1997). The search for life on Mars. Orig Life Evol Biosph.

[pbio-0020302-Mileikowsky1] Mileikowsky C, Cucinotta FA, Wilson JW, Gladman B, Horneck G (2000). Natural transfer of viable microbes in space. Icarus.

[pbio-0020302-Pace1] Pace N (2001). The universal nature of biochemistry. Proc Natl Acad Sci U S A.

[pbio-0020302-Raven1] Raven JA, Kübler JE, Beardall J (2000). Put out the light, and then put out the light. J Mar Biol Ass (UK).

[pbio-0020302-Schrodinger1] Schrödinger E (1945). What is life?.

[pbio-0020302-Stevens1] Stevens TO, McKinley JP (1995). Lithoautotrophic microbial ecosystems in deep basalt aquifers. Science.

[pbio-0020302-Ultee1] Ultee A, Souvatzi N, Maniadi K, König H (2004). Identification of the culturable and nonculturable bacterial population in ground water of a municipal water supply in Germany. J Appl Microbiol.

[pbio-0020302-Weiss1] Weiss BP, Kirschvink JL, Baudenbacher FJ, Vali H, Peters NT (2000). A low temperature transfer of ALH84001 from Mars to Earth. Science.

[pbio-0020302-Woese1] Woese CR (1987). Bacterial evolution. Microbiol Rev.

